# Head and Whisker Behaviours Observed During Foraging in Northern Elephant Seals (*Mirounga angustirostris*)

**DOI:** 10.1002/ece3.73684

**Published:** 2026-05-17

**Authors:** Morgan Chapman, Héloïse Frouin‐Mouy, Fabio C. De Leo, Robyn A. Grant

**Affiliations:** ^1^ Department of Natural Sciences Manchester Metropolitan University Manchester UK; ^2^ Cooperative Institute for Marine and Atmospheric Studies University of Miami Miami Florida USA; ^3^ Department of Biology University of Victoria Victoria British Columbia Canada; ^4^ Ocean Networks Canada University of Victoria Victoria British Columbia Canada

## Abstract

Pinnipeds use their whiskers to detect and follow hydrodynamic cues during foraging. The movement of their head and whiskers is likely to be important for positioning the whiskers towards salient points in the flow‐scape. We present here the first descriptions of whisker and head movements in freely moving, foraging male northern elephant seals ( *Mirounga angustirostris* ) using a novel, non‐invasive underwater filming set‐up. We observed a range of behaviours during foraging, including forward swimming, vertical diving, diving with rotations, swooping swimming motions, vertical head dabbing (or bobbing), and lateral head sweeping, which occurred at different timescales, ranging from long, continuous swims to 0.4 s dabs. These different types of movements in time and space likely provide the seals with the range of head and whisker positions and timings necessary for successful foraging. We also observed prolonged whisker protractions and rhythmic whisker movements with amplitudes of 10°–130°. These are the largest ever measured in pinnipeds. It is likely that only by observing naturalistic behaviours, such as foraging in the wild, can these large, full whisker movements be observed. With this in mind, continuing to develop technology to observe naturalistic whisker movements in the field will give us better insights into the sensory behaviours of pinnipeds and other mammals.

## Graphical abstract

We observed a range of behaviours during foraging, including forward swimming, vertical diving, swooping, dabbing, sweeping and rotations. Head dabbing was also sometimes accompanied by the seal producing infrasounds, the bouts of which coincided with rhythmic whisker protractions.

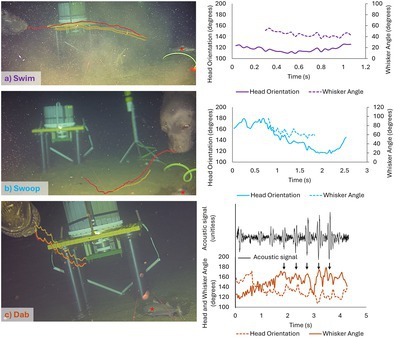

## 1 Introduction

Many pinnipeds hunt in deep, turbid waters, and rely on their whiskers (or vibrissae) to guide prey detection in these dark environments (Dehnhardt et al. 2014). Specifically, pinnipeds can use their whiskers to detect hydrodynamic cues produced by moving prey (Grant 2025a; Grant and Goss 2022). Indeed, behavioural training tasks in captive harbour seals ( *Phoca vitulina* ) have shown that they use their whiskers to detect artificial flatfish breathing currents (Niesterok et al. 2017), discriminate different wakes (Wieskotten et al. 2011), as well as to trail‐follow submarines (Wieskotten et al. 2010b) and conspecifics (Schulte‐Pelkum et al. 2007). Movement is an important part of whisker sensing (Grant 2025a), as well as touch tasks more generally (Gibson 1962; Lederman and Klatzky 1987). The movement of the head and whiskers will undoubtedly affect the resulting sensations of the whiskers; therefore, movement and perception are closely coupled in whisker sensing.

During hydrodynamic trail‐following, seals can either directly follow a trail in a linear fashion, or undulate across the trail, repeatedly crossing it (Schulte‐Pelkum et al. 2007). Due to the relatively large movements of the head, compared to the whiskers, it was previously thought that head movements, rather than whisker movements, were more important for ensuring efficient whisker positioning during use (Dehnhardt 1994; Grant et al. 2013). In addition, protracting the whiskers underwater likely carries a high energetic cost, due to the added drag of the water (Adachi et al. 2022). However, underwater protractions have been observed during a touch task in harbour seals (Grant et al. 2013), and they have a large, specialised architecture of muscles to enable underwater protractions (Elder, Evans, et al. 2025). Up until very recently, pinniped whiskers had only been studied using captive collections (Grant 2025a). However, a recent study by Adachi et al. (2022) captured the hydrodynamic foraging patterns of pinnipeds in the wild for the first time. Specifically, the authors applied head‐mounted video cameras and accelerometers to female northern elephant seals ( *Mirounga angustirostris* ) and investigated their foraging behaviours. They observed rhythmic whisker movements (Adachi et al. 2022), the likes of which had only previously been observed in terrestrial mammals (Muchlinski et al. 2020), and were assumed to be absent in aquatic species. Indeed, rhythmic whisker movements, or whisking, can be observed across mammals, and may have evolved independently as many as eight times across the clades Marsupialia, Afrosoricida, Eulipotyphla, Rodentia (Muchlinski et al. 2020), and now also in the Carnivora. Adachi et al. (2022) suggested that rhythmic and prolonged whisker protractions are key to detecting, pursuing, and capturing prey, likely by positioning the whiskers towards salient points within the flow‐scape.

Unfortunately, due to the positioning of the cameras, the head orientation and head movements could not be captured by Adachi's study. However, the coupling of head and whisker movements is an important aspect of orienting, including head rotations with whisker asymmetry (termed head‐turning asymmetry) (Grant et al. 2023) and vertical head movements with whisker protractions (termed dabbing) (Grant et al. 2012, 2013). Measuring the whiskers relative to the head would, therefore, be beneficial. Whisker movement amplitudes, relative to the head, of another phocid, the harbour seal, can range from 17° to 28° in touch tasks (Grant et al. 2023; Milne et al. 2020), and it would be interesting to see how the Northern elephant seals compare to these.

Recently, a novel field‐based study described the behaviour of male Northern elephant seals from video footage positioned at a deep‐sea cabled observatory (Frouin‐Mouy et al. 2024). While not specifically measuring head or whisker movements, the authors observed much activity during foraging, including searching, swimming and pursuing prey. Video footage collected in this way would offer the ability to observe and measure both head and whisker movements during natural foraging, which has never been done before. Therefore, our study adopts a video set‐up established at a deep‐sea cabled observatory (Frouin‐Mouy et al. 2024) to investigate whether it can feasibly be used to observe and measure head and whisker movements in male Northern elephant seals. Collected footage will first be used to qualitatively describe head and body movements during foraging, and then to track the head and whisker positions. Overall, we would expect to see large head movements positioning the whiskers, as well as rhythmic whisker movements, such as those first described by Adachi et al. (2022). Evidence of which would emphasise the importance of active whisker sensing during natural foraging in pinnipeds.

## 2 Methods

### 2.1 Study Site

This study makes use of the video data generated in Frouin‐Mouy et al. (2024) using Ocean Networks Canada's (ONC) large, regional cabled observatory network. The North‐East Pacific Time‐series Undersea Networked Experiments (NEPTUNE) observatory includes an 800‐km network of subsea fibre optic cables connecting multiple seafloor nodes that provide power and bandwidth to thousands of sensors. The study site (48°34′57.9″ N, 126°15′77.1″ W) was located ~100 km offshore from Barkley Sound, Vancouver Island, at a depth of 645 m, and is one of these nodes. Barkley Canyon Node was the selected site for the installation of a Fish Acoustics and Attraction Experiment (FAAE) designed to combine observations from video, acoustic imaging sonar, and underwater sounds from a hydrophone, to examine the effects of light, bait introduction and background noise on fish and invertebrate behaviours. The bait release system was programmed to release one sardine, preserved in vegetable oil, every 14 days during the study period. Bait release times were synchronised so they would be captured during video recording. An HD video camera was positioned on a platform, 63 cm above the seabed with an image area of ~12 m^2^ [see Frouin‐Mouy et al. (2024) for more information]. Videos were gathered from 21 May 2022 to 16 July 2023 (422 days). In total, 9737 ~5‐min clips (~811 h) were collected during this period and elephant seals were observed in 113 videos. All observations were of male, sub‐adult elephant seals, and eight individuals were identified using visible body marks or scars, as well as eyeliner cues (Frouin‐Mouy et al. 2024). Identified individuals ranged from 4 to 7 years. Adult males are known to travel to coastal areas and forage on the continental shelf, including off Washington state and British Columbia (Kienle et al. 2022; Le Boeuf et al. 2000). However, the presence of only sub‐adult males near Barkley Canyon Node (Frouin‐Mouy et al. 2024), a site closer to their rookeries (from central Baja California to Oregon), may be explained by differences in the timing of foraging trips between sub‐adult and adult males, and/or a higher likelihood of encountering sub‐adult males due to the greater mortality rates observed in adult males (Clinton and Le Boeuf 1993; Kienle et al. 2022).

Observations of northern elephant seals did not require permits since the cameras were remotely placed (underwater) and did not cause disturbance to the animals. Ethics were approved by the local ethics committee at Manchester Metropolitan University (ID: 81660). It should be noted that at the time of the FAAE experimental deployment, ONC and the principal investigators were not aware that an Animal Use Protocol (AUP) was required as part of the ethics approval process by University of Victoria's Animal Care Committee (ACC). The ACC uses the Canadian Council on Animal Care (CCAC) definition of sentient animals, that is all non‐human vertebrates and cephalopods and, since the FAAE experiment employed baiting (described in Frouin‐Mouy et al. 2024), it had the potential to attract vertebrates and cephalopods.

### 2.2 Video Analysis

**FIGURE 1:**
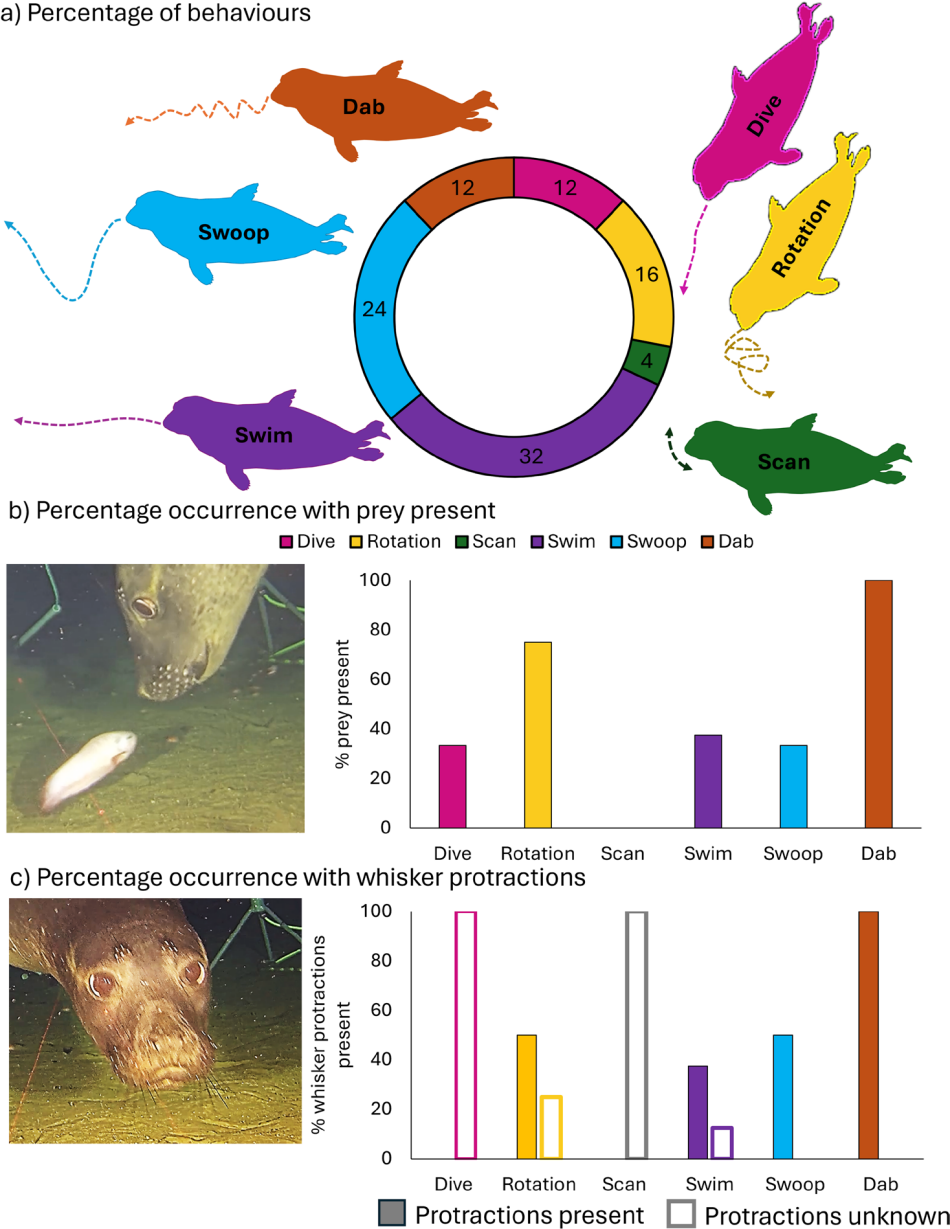
Percentages of behaviours observed in 25 clips of foraging northern elephant seals. (a) Swimming was the most common behaviour captured, followed by swooping, rotating, diving and dabbing. Scanning was only observed in one video. Video tracking could not be undertaken on behaviours that were out of plane in the video; therefore further analysis was conducted only on swimming, swooping and dabbing behaviours, representing 68%, or 15 of the 25 clips captured. (b) Prey items were present in all dabbing behaviour clips, and most rotation behaviour clips. No prey items were observed during the one scanning clip. (c) Protractions were present in all dabbing behaviour clips and 50% of rotation and swooping clips. Whiskers could not be clearly observed to assess protractions in diving and scanning clips. Example video screenshots of our male, sub‐adult elephant seals. Panel b screenshot shows a fish prey item with a seal [named Dennis and featured in figure 4 in Frouin‐Mouy et al. (2024)], and panel c screenshot shows protractions of the whiskers of a seal [named Brian and featured in figure 4 in Frouin‐Mouy et al. (2024)].

**FIGURE 2:**
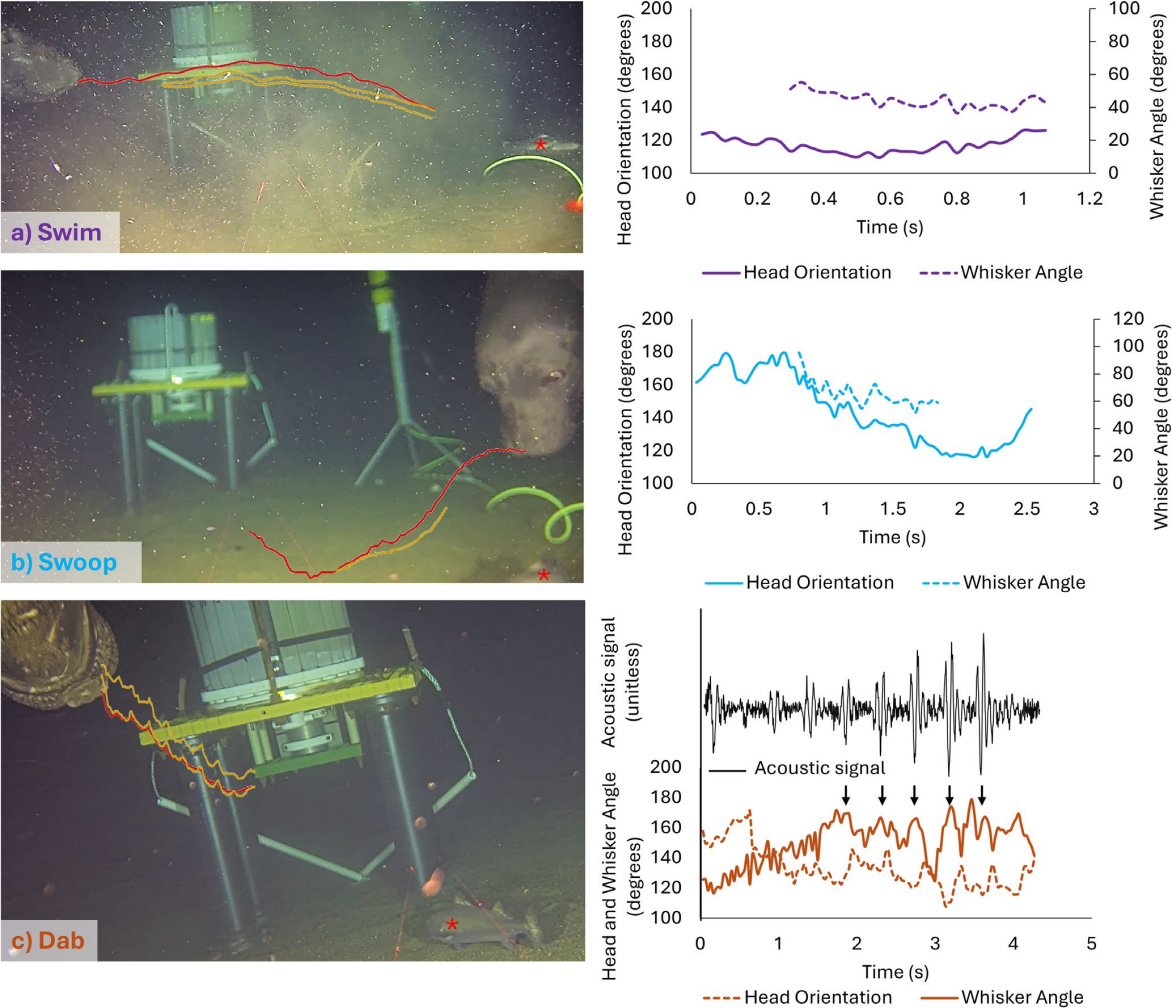
Example tracking (left) alongside extracted head and whisker angles from male, sub‐adult northern elephant seals (right). In the left panels, nose tip tracking is in red, and whisker shaft tracking points are in yellow. Prey items are identified by the red asterisk (*). Two whiskers were tracked for the swimming and dabbing examples, and only one in the swooping example. In the right panels head angular orientation is shown by the full line, and the mean angle of all tracked whiskers is shown by the dotted line, for swimming (in purple), swooping (in blue) and dabbing (in orange). An amplified (unitless) acoustic signal (in black) is also included, which reveals an infrasonic sound that the seal made at the same time as head dabbing (in orange). Black arrows correspond to the acoustic signal burst coinciding with peaks in rhythmic whisker protractions.

All 113 videos were reviewed to include instances where the head and whiskers were clearly visible for > 1 s and the seal was far enough away from the camera for their foraging behaviours to be clearly observed through the clip. This led to 25 clips being included for further analysis. Foraging behaviours were identified in each of these clips. They were described in an ethogram table (Table 1) and pictorially in Figure 1a. Where possible, the durations of discrete aspects of these behaviours were recorded from each clip by manually reviewing the footage. The presence of a prey item that the seal was pursuing was also noted (Figure 1b, and indicated by a red asterisk in Figure 2), as well as whether whisker protractions were present, absent or could not be assessed (termed unknown, which occurred in six clips) (Figure 1c). Clips were then further reviewed for head and whisker tracking. Clips were further discounted if: (i) the head or whiskers were unclear or out of view, or (ii) there was significant ‘out of plane’ head movements to the camera. In total, 15 video clips were manually tracked using the Manual Whisker Annotator (Hewitt et al. 2016). The nose tip and mid‐snout position were tracked in each frame to extract head orientation (Figure 2). One to three whiskers were tracked on one side of the face in each clip, depending on how many whiskers were clearly visible. Only one side could be tracked in the videos, so there was no bilateral tracking and whisker asymmetry could not be extracted. One point on the base and one point on the whisker shaft was tracked in each frame, and an angle was calculated between the whisker and head orientation, giving that larger angles indicated more forward positioned whiskers and smaller angles indicated more backward positioned whiskers. A mean whisker angle (Figure 2) was calculated across the tracked whiskers, since the whiskers tended to move together. Head and whisker summary metrics were then calculated, including head and whisker amplitude (the maximum minus the minimum head and whisker angles), and whisker angle (the mean angle of all whisker angles). These metrics were compared between different behaviours, and in the presence and absence of prey items, using Kruskal–Wallis non‐parametric tests, which were chosen due to the small sample numbers. Indeed, due to the opportunistic nature of video collection, the total number of videos is low. Therefore, it must be stated that the results presented below represent exploratory descriptions of the video footage collected, and further behaviours and quantifications are likely to be present in other, future video examples.

**TABLE 1:** Description of observed behaviours in 25 video clips of foraging elephant seals.

Behaviour	Definition
Dive	Swimming downwards towards ground
Rotation	Diving down with clear corkscrew twist or turn, out of plane
Scan	Right and left scanning of head during horizontal swimming, out of plane
Swim	Horizontal swimming
Swoop	Small diving movement, followed by an upward movement, can also be observed in reverse (upward, followed by downward movement), taking > 1 s in duration
Dab	Repetitive, rhythmic head movements up and down, with each cycle taking < 1 s (~0.5 s)

In one tracked video example, an infrasonic sound was also made by the seal. This acoustic sound signal was extracted from hydrophone recordings at the site and processed. This included removing the mean amplitude (to remove the DC offset), normalising, low‐pass filtering (100 Hz), applying a pitch shifting algorithm (iZotope Radius), and amplifying the signal to make it audible. It was then calibrated in time to the tracked video footage and plotted alongside, as a unitless signal.

## 3 Results

### 3.1 Qualitative Description of Foraging Behaviours

Six clear behaviours were observed from the footage and were defined in Table 1. Their percentages of occurrence and a descriptive diagram of the behaviour can be seen in Figure 1. Swimming involved horizontal swimming across the camera, with minimal vertical movements (Figure 2a), and was observed in eight clips. Vertical movements downwards included a dive (a long duration behaviour involving swimming downwards to the seabed), which was observed in three clips. Rotations involved a dive or horizontal swim, with a tight twist or turn. These were observed in four clips, and a whole rotation took ~3.5 s (3.4–4.0 s). Scanning was observed in only one clip, with left to right movements of the head, occurring at scan durations of ~3 s (2.3–3.1 s). Head swooping involved a head dip down or up, during horizontal swimming, durations of each swoop or dip took around 2.5 s (Figure 2b) (ranging from 1.7 to 4 s) and was observed in six clips. Dabbing involved a quicker vertical head bob, which took around 0.4 s for each dab (Figure 2c) (ranging from 0.4 to 0.5 s), and was observed in three clips.

**FIGURE 3:**
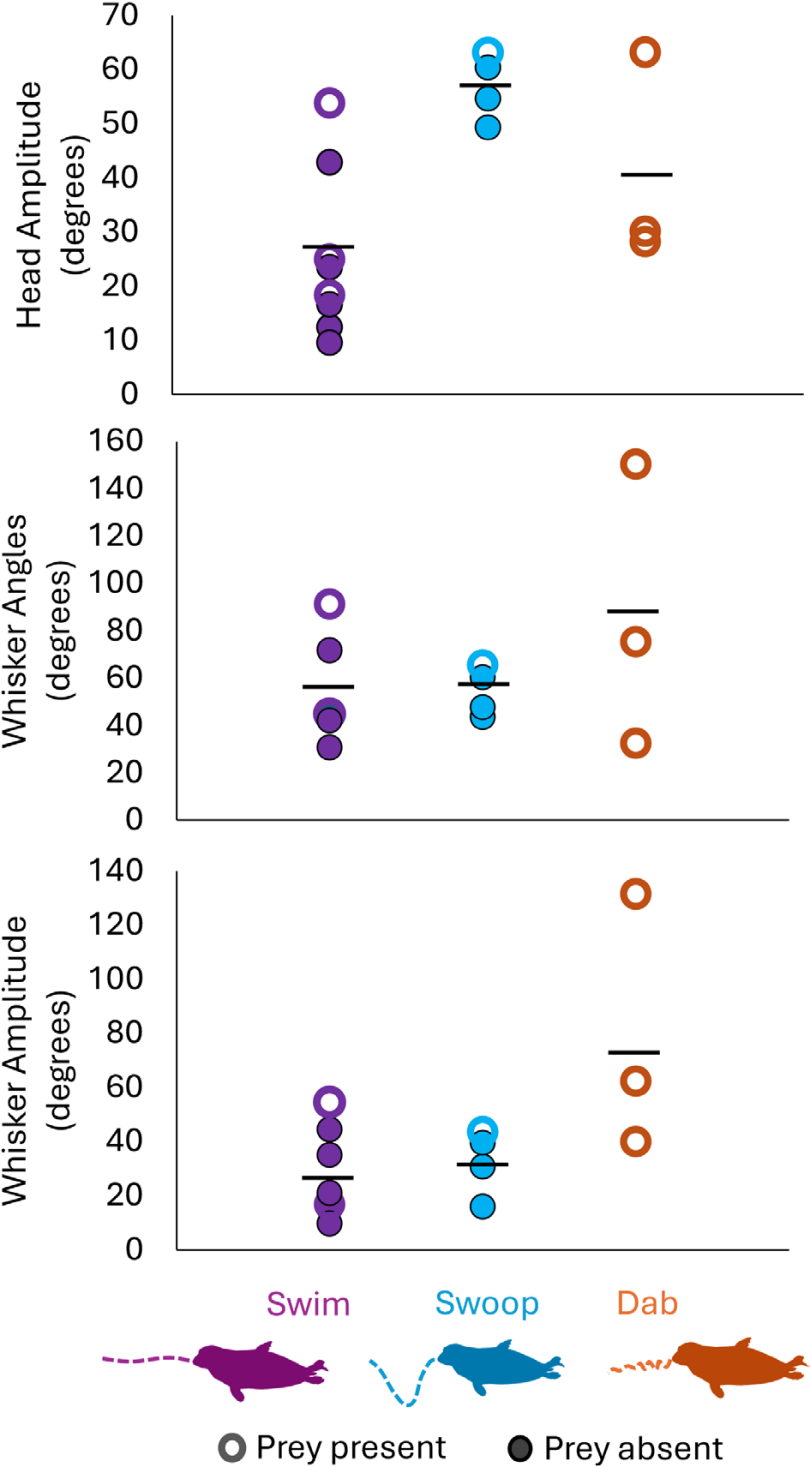
Head and whisker movements of the seals during swimming (purple, *n* = 8), swooping (blue, *n* = 4) and dabbing (orange, *n* = 3) behaviours. Swooping had significantly larger head amplitudes. Whisker angles and amplitudes did not vary significantly between behaviours, but whisker amplitudes tended to be higher during dabbing. The presence of prey (unfilled marker) did not affect any of the head and whisker metrics. Individual markers correspond to each tracked clip, black lines correspond to the mean value of all individual markers.

Prey items were observed in 11 (44%) of the video clips, including fish (*n* = 10, 91%) and octopus (*n* = 1, 9%). They were always present when the seal performed dabbing behaviours, and usually present when the seal performed rotation behaviours (Figure 3b). Otherwise, they were absent during the scanning clip and present in 30%–40% of clips in other behaviours (Figure 3b). Whisker protractions were present in 11 (44%) of the video clips and absent in 8 clips (32%) and could not be assessed in 6 (24%) clips. Whisker protractions were always present when the seal performed dabbing behaviours, and in 50% of the rotation and swooping clips.

### 3.2 Head and Vibrissal Tracking

Whisker and head tracking could only be undertaken in 15 clips involving swimming, swooping and diving (Figure 2a) due to significant out of plane movements involved in the clips of other behaviours. Head amplitude was significantly larger during swooping movements, compared to dabbing and swimming (Kruskal–Wallis: *H* = 7.519, df = 2, *p* = 0.023) (Figure 3). There were no significant differences in whisker angles or amplitudes between the behaviours (whisker angle Kruskal–Wallis: *H* = 0.769, df = 2, *p* = 0.681; whisker amplitude Kruskal–Wallis: H = 4.856, df = 2, *p* = 0.088), although whisker amplitudes did tend to be larger during dabbing behaviours (~40°–130°), compared to during swimming (~30°–90°) or swooping (~15°–45°) behaviours. Indeed, these can be seen as clear, rhythmic whisker movements in Figure 2c. These whisker movements coincided with the head dabs, every ~0.4 s, with whiskers moving forwards as the head moves down. The presence of a prey item did not affect whisker angles, amplitudes or head movements (all *p*s > 0.05) (compare filled and open points in Figure 3).

## 4 Discussion

A range of different head and body movements were observed from the elephant seals during foraging, including swimming, diving, swooping, dabbing, sweeping and rotations (Video 1). These movements occurred at different timescales, ranging from long, continuous movements (swimming), head rotations, swooping and scanning (taking 1.7–4 s) and dabbing (taking 0.4–0.5 s per dab). While head movements were large and prominent, whisker movements were also observed relatively commonly. Where whisker movements could be assessed, they were present in the majority of all clips (almost 60% or 11/19 of the clips where whisker movements could be assessed), and rhythmic whisker movements were also observed during head dabbing. The quickest head and whisker movements (dabbing) only occurred when prey was present. These different types of movements in time and space likely give the seals the correct range of head and whisker movement positions and timings that are needed for successful foraging (Video 1). The positioning of the whiskers into salient flow spaces in the environment is likely an integral part of hydrodynamic sensing, and timing will also be important during prey pursuit.

**VIDEO 1:** Head and whisker behaviours observed during foraging in elephant seals. Example videos of whisker protractions movements, swimming, diving, rotations, swooping, scanning and dabbing. Dabbing can be accompanied by infrasounds too. All these behaviours come together to help elephant seals forage. Video content can be viewed at https://onlinelibrary.wiley.com/doi/10.1002/ece3.73684.

In agreement with Adachi et al. (2022), we observed prolonged whisker protractions in the majority of our video clips. We cannot directly compare our whisker measurements to theirs, since we measured whiskers using the angle the whiskers make with the head (as per Grant et al. 2013, 2023; Milne et al. 2020, 2021; Milne and Grant 2014; Nakhwa et al. 2024), which could not be approximated from the head‐mounted video camera view of Adachi's study. Whisker protractions have been previously observed, although not measured, in foraging pinnipeds (Dehnhardt et al. 2001, 2014; Gläser et al. 2011; Schulte‐Pelkum et al. 2007; Wieskotten et al. 2010a) and will play a role in orienting the whiskers towards hydrodynamic stimuli. Whisker movements (amplitudes) in the elephant seals were large, ranging from retractions that were fully flat to cheek (~30°) to fully protracted at 150°, giving amplitudes of 10°–130°. These are among the largest ever measured in pinnipeds. Indeed, harbour seal whisker amplitudes have been found to be 17°–28° (Grant et al. 2023; Milne et al. 2020), South African fur seals (*Acrtocephalus pussilus*) to be 32°–36° (Nakhwa et al. 2024) and California sea lions ( *Zalophus californianus* ) to be around 20°–120° (Milne and Grant 2014; Milne et al. 2020) during touch tasks. It might be that hydrodynamic whisker‐use requires larger whisker movements, and we have not seen such big movements before since whisker movements have never been measured in a hydrodynamic task. However, it is probably more likely that, while enrichment tasks have been developed to try to encourage larger and more natural whisker movements (Elder, Todd, et al. 2025; Milne and Grant 2014; Milne et al. 2020; Nakhwa et al. 2024), only during purely naturalistic behaviours, such as foraging in the wild, can these large, full whisker movements be observed (Grant 2025a).

Similar to Adachi et al. (2022), we also observed rhythmic whisker movements (Figure 2c), although these were much faster (< 1 s) than Adachi's observations, where whisker cycles took around 9 s. The rhythmic whisker movements we observed coincided with head dabs, where the protraction phase of the whisker movements occurred at the same time as the downward movement of the head. Whisker movements occurring with head dabs have been characterised in rats (Grant et al. 2012), and also observed in harbour seals (Grant et al. 2013). Moving the head and whiskers together facilitates orienting of the whiskers and head to the same place at the same time (Grant et al. 2012). Frouin‐Mouy et al. (2024) observed that these dabbing movements also often coincided with infrasonic sounds (~12 Hz) and that the dabbing head movements may be used to generate the sounds, either voluntarily or involuntarily. Indeed, we also observed an infrasonic sound in one of our tracked dabbing videos (Figure 2c, Video 1), and the bursts of acoustic signal appear to coincide with peak rhythmic whisker protractions (indicated by the arrows in Figure 2c). Perhaps producing the infrasonic noise uses the same extrinsic facial muscles that protract the whiskers, since the muscles that control the mouth and whiskers are very similar in Phocids (Elder, Evans, et al. 2025). That the sound is present alongside rhythmic head and whisker movements, might suggest that the seal makes sound vibrations in the water to supplement its hydrodynamic sensing techniques, similar to echolocation. Renouf et al. (1980) first proposed that seals might echolocate, and observed harbour seals making sounds of 6–60 Hz, especially at night in the dark (values extracted from figure 1 in Renouf et al. (1980) and figure 1b in Renouf and Davis (1982)). Our observed sound falls within this frequency range. Renouf et al. (1980) suggest that the produced sound may be used in a similar way to echolocation to guide the seal towards a prey item, and then the whiskers would take over as proximate sensors when the fish is close enough to guide the attack. Alternatively, the sound might be used to startle prey, since locating moving prey might be easier. Sound and vibrissal sensing has not yet been investigated in pinnipeds, although they may well be able to detect sound with their whiskers, in addition to touch and hydrodynamic signals (Grant 2025a, 2025b). Indeed, Saimaa ringed seals have a distinct fatty acid composition around vibrissal follicles, which is different to that of blubber (Käkelä and Hyvärinen 1993) and has been suggested to translate sound signals and improve acoustic sensing using the whiskers (Käkelä and Hyvärinen 1993). Our observations in this study are only based on one single event, where we could clearly track the head and whiskers. However, Frouin‐Mouy et al. (2024) observed 11 instances of head dabbing, with 10 (91%) of these instances accompanied by infrasonic sound. Therefore, the role of acoustic cues during foraging, and especially in conjunction with vibrissal sensing, should be investigated further, since it may have interesting implications for active foraging, as well as whether it can be affected by noise pollution (Grant 2025b).

Using our novel set‐up at a deep‐sea cabled observatory we are able to observe and take precise measurements of male elephant seal head, whisker and body movements, and start to characterise specific foraging behaviours for the first time (Video 1). The observatory may have influenced the seal presence and behaviour somewhat, including through the introduction of acoustic noise, light and bait. We may expect seals to rely on their whiskers even more in darker environments away from the observatory, where artificial light is absent. The addition of bait to the observatory likely attracted the fish prey observed during hunting events. Indeed, we observed different head movements and foraging behaviours operating over different spatial and temporal scales, and some of the largest whisker movements ever documented. However, the video recordings are only opportunistic, and the resolution and frame rate of the cameras make it not possible to track the whiskers in many of the clips. This means that the sample numbers are low in this study. Nevertheless, this study still gives us new insights into pinniped foraging behaviours. In addition, the development of technology and sensors are continually improving and are set to produce higher quality and faster data into the future (Grant 2025a). Continuing to develop this technology to observe naturalistic whisker movements in the field will give us better insights into the sensory behaviours of pinnipeds and other mammals.

## Author Contributions

**Morgan Chapman:** data curation (equal), formal analysis (equal), methodology (equal). **Héloïse Frouin‐Mouy:** conceptualization (equal), data curation (equal), formal analysis (equal), investigation (equal), methodology (equal), project administration (equal), supervision (equal), writing – review and editing (equal). **Fabio C. De Leo:** conceptualization (equal), data curation (equal), formal analysis (equal), supervision (equal), visualization (equal), writing – review and editing (equal). **Robyn A. Grant:** conceptualization (equal), formal analysis (equal), investigation (equal), supervision (equal), validation (equal), visualization (equal), writing – original draft (equal), writing – review and editing (equal).

## Funding

This work was supported by Canada Foundation for Innovation (53000).

## Conflicts of Interest

The authors declare no conflicts of interest.

## Supporting information


**Data S1:** ece373684‐sup‐0001‐DataS1.zip.

## Data Availability

All data used in this manuscript can be found in the Supporting Information file. Hydrophone data used in this study, and many other additional examples, are freely available at https://data.oceannetworks.ca/SearchHydrophoneData, and the processed hydrophone data can be found in the Supporting Information file. All videos can be shared on request.
