# Supplementary material for: Head and Whisker Behaviours Observed During Foraging in Northern Elephant Seals (Mirounga angustirostris)
Source: Ecol Evol. 2026 May 17;16(5):e73684. doi: 10.1002/ece3.73684 (PMC13180480; doi:10.1002/ece3.73684)
Supplement: Supplementary file 2 — Data S1: ece373684‐sup‐0001‐DataS1.zip. [file ECE3-16-e73684-s001.zip › ece373684-sup-Supplementary.pdf]

Supplementary Data 1: Scored video clips

ClipName	Tracked	behaviour	fish	whisker protractions	Mean angle	Amp	head amp
20221228-0001	1	swoop	0	0	43.74	15.87	49.28
20220724-0001	1	swoop	0	1	47.69	30.71	60.36
20220623-0002	1	swoop	0	1	60.29	39.52	54.59
20220622-0001	1	swoop	1	1	65.43	43.62	63.12
20220905T1-0002	1	swim	0	1	71.87	44.35	12.39
20220905T21-0001	1	swim	0	0	30.76	35.04	23.47
20221010-0002	1	swim	0	0	44.40	18.84	16.59
20220704-0001	1	swim	1	1	44.91	54.47	53.80
20221010-0001	1	swim	1	1	44.29	14.98	24.97
20220628-0002	1	swim	0	0	71.83	9.77	9.51
20220627-0001	1	swim	1		91.22	16.70	18.30
20221008-0001	1	swim	0		42.01	20.98	42.89
20221009-0001	0	scanning	0				
20221001-0001	0	rotation	1				
20221010-0001	0	rotation	1				
20221219-0004	1	dab	1		32.52	39.99	28.15
20230515-0001	1	dab	1		150.38	62.33	63.29
20220724-0002*	1	dab	1 attack		75.28	131.54	30.13
20221005-0001-1	0	swoop	0	1			
20221010-0001	0	rotation	1	1			
20220704T021-0001	0	swoop	1	1			
20221008-0002	0	swim	0				
20221219-0001	0	dive	0				
20221219-0002	0	dive	0				
20221219-0003	0	rotation	1				

* featured in supplementary video

Supplementary Data 2: Featured video tracking in plots

Bob Example – 20230515-0001

Time	Index	Head Orientation	Whisker 1	Whisker 2	mean
0.033333	1	157.8906	139.4556	112.1094	125.7825
0.066667	2	149.6419	128.9163	122.9029	125.9096
0.1	3	149.9314	116.7529	120.0686	118.4107
0.133333	4	147.2251	120.6601	126.3512	123.5056
0.166667	5	150.6039	110.2394	122.8297	116.5345
0.2	6	153.7587	121.9312	117.4867	119.709
0.233333	7	153.7334	124.1946	117.512	120.8533
0.266667	8	150.9454	115.6342	124.8809	120.2576
0.3	9	152.4472	138.8768	119.7986	129.3377
0.333333	10	156.8014	138.3665	116.7749	127.5707
0.366667	11	160.796	136.1725	107.9309	122.0517
0.4	12	162.8544	152.0702	110.9596	131.5149
0.433333	13	165.9638	143.3439	106.1573	124.7506
0.466667	14	165.2803	160.7064	107.8418	134.2741
0.5	15	164.1886	148.2432	107.8568	128.05
0.533333	16	165.1317	153.8218	104.8683	129.345
0.566667	17	167.0822	164.0694	100.7152	132.3923
0.6	18	166.8491	179.3779	104.4529	141.9154
0.633333	19	171.1354	146.1185	98.86462	122.4916
0.666667	20	145.9807	138.8556	146.4988	142.6772
0.7	21	146.9207	143.3444	133.5707	138.4576
0.733333	22	144.2602	137.5504	121.3411	129.4457
0.766667	23	141.2773	141.2773	126.4321	133.8547
0.8	24	141.1269	153.6557	123.5585	138.6071
0.833333	25	139.9166	157.2706	132.9458	145.1082
0.866667	26	141.6913	171.9477	129.7775	150.8626
0.9	27	135.9935	130.799	134.0065	132.4028
0.933333	28	141.8144	160.2493	131.3655	145.8074
0.966667	29	143.8264	141.3368	135.336	138.3364
1	30	143.6156	134.1533	126.3844	130.2688
1.033333	31	139.0856	135.7192	151.3243	143.5217
1.066667	32	135.3274	146.6373	147.2014	146.9194
1.1	33	128.8845	139.6688	149.8617	144.7652
1.133333	34	129.6107	132.9771	160.9454	146.9613
1.166667	35	131.236	136.2057	164.7384	150.4721
1.2	36	122.9977	129.7075	155.6548	142.6812
1.233333	37	130.1009	130.1009	149.9797	140.0403
1.266667	38	132.1579	135.3377	157.9374	146.6375
1.3	39	132.1579	145.1525	157.9374	151.5449
1.333333	40	135.8551	126.8825	157.1704	142.0264

1.366667	41	128.9275	131.5301	175.8503	153.6902
1.4	42	128.6598	136.2545	173.2062	154.7303
1.433333	43	129.4007	125.5866	164.5618	145.0742
1.466667	44	136.0544	148.3192	165.5531	156.9361
1.5	45	134.3839	129.4142	147.5047	138.4595
1.533333	46	130.6568	154.8845	160.4481	157.6663
1.566667	47	127.1847	169.0049	155.5728	162.2889
1.6	48	126.1075	177.1799	147.9782	162.579
1.633333	49	130.1696	173.5205	146.0339	159.7772
1.666667	50	127.4606	179.4093	148.1116	163.7605
1.7	51	122.9052	169.1783	159.899	164.5386
1.733333	52	124.1436	175.1526	168.1054	171.629
1.766667	53	124.804	179.9795	157.5767	168.7781
1.8	54	122.4211	170.7875	156.1097	163.4486
1.833333	55	126.076	176.2704	161.5164	168.8934
1.866667	56	129.6442	178.4029	160.6384	169.5206
1.9	57	133.1524	176.8202	161.7915	169.3058
1.933333	58	145.2651	177.865	141.2919	159.5784
1.966667	59	142.8291	177.6113	133.1178	155.3645
2	60	142.5684	171.2127	143.872	157.5424
2.033333	61	136.2545	174.5606	142.0427	158.3017
2.066667	62	135	173.6598	143.1301	158.395
2.1	63	137.7263	159.7481	135.4535	147.6008
2.133333	64	136.1458	166.1299	138.4897	152.3098
2.166667	65	131.2686	173.4266	147.0973	160.2619
2.2	66	135	165.9638	146.3099	156.1368
2.233333	67	135	168.6901	150.2551	159.4726
2.266667	68	132.337	169.6684	156.441	163.0547
2.3	69	131.6335	170.6421	163.2302	166.9361
2.333333	70	133.6028	168.4026	158.4085	163.4055
2.366667	71	143.3103	165.0572	148.7259	156.8916
2.4	72	145.4077	165.7782	144.0947	154.9365
2.433333	73	133.7078	176.8602	150.913	163.8866
2.466667	74	128.1076	171.3683	146.7569	159.0626
2.5	75	125.3625	159.2584	154.9424	157.1004
2.533333	76	128.7844	153.1043	140.2916	146.6979
2.566667	77	125.6279	159.8962	137.9305	148.9133
2.6	78	124.6952	169.7488	135.6851	152.717
2.633333	79	123.6901	161.834	134.3658	148.0999
2.666667	80	123.0239	168.0136	150.0983	159.056
2.7	81	120.0686	164.9999	162.1029	163.5514
2.733333	82	121.2637	167.7619	163.3105	165.5362
2.766667	83	122.0054	170.6145	161.5305	166.0725
2.8	84	126.0274	169.742	151.2476	160.4948

2.833333	85	133.3317	169.2876	145.7078	157.4977
2.866667	86	138.3665	167.7917	127.733	147.7624
2.9	87	132.7094	157.2737	111.8829	134.5783
2.933333	88	129.2894	164.2092	103.8407	134.0249
2.966667	89	130.684	153.8505	101.7474	127.799
3	90	125.3401	147.8397	102.3024	125.0711
3.033333	91	124.1597	155.6227	135.9141	145.7684
3.066667	92	121.9081	150.213	139.1193	144.6661
3.1	93	114.7174	168.7783	151.1969	159.9876
3.133333	94	107.8503	171.612	152.6874	162.1497
3.166667	95	110.2249	174.3096	164.0643	169.1869
3.2	96	110.8978	178.6389	169.8865	174.2627
3.233333	97	118.0245	176.5991	165.216	170.9075
3.266667	98	126.4692	169.1718	150.4837	159.8277
3.3	99	128.3675	166.5373	151.0948	158.8161
3.333333	100	134.5342	156.9672	125.4578	141.2125
3.366667	101	128.5505	160.7396	163.653	162.1963
3.4	102	120.1013	158.0288	163.1392	160.584
3.433333	103	118.8108	174.3023	170.1139	172.2081
3.466667	104	117.6995	178.8656	178.8656	178.8656
3.5	105	122.6609	163.7286	174.7467	169.2376
3.533333	106	122.2288	144.7585	161.4425	153.1005
3.566667	107	120.7841	156.6473	155.122	155.8846
3.6	108	115.56	168.4763	160.3462	164.4112
3.633333	109	117.646	165.7465	168.8584	167.3025
3.666667	110	117.6995	163.1861	167.127	165.1565
3.7	111	128.8845	161.4386	155.1517	158.2952
3.733333	112	135.6296	151.896	138.3613	145.1286
3.766667	113	129.1304	162.671	145.0545	153.8628
3.8	114	120.6507	163.3856	156.4743	159.93
3.833333	115	121.4768	157.9855	159.2363	158.6109
3.866667	116	120.3432	157.0882	160.3699	158.729
3.9	117	120.5297	155.1809	157.6004	156.3906
3.933333	118	119.5778	156.1328	159.6948	157.9138
3.966667	119	117.8973	160.5297	156.8664	158.698
4	120	115.9065	163.1838	163.0661	163.1249
4.033333	121	115.821	164.3037	167.806	166.0548
4.066667	122	116.5651	172.4606	166.2392	169.3499
4.1	123	125.2176	167.981	155.3216	161.6513
4.133333	124	133.8065	162.7585	147.5034	155.131
4.166667	125	134.3969	152.3023	157.671	154.9867
4.2	126	130.4261	162.1938	139.5739	150.8839
4.233333	127	131.9872	156.4477	138.0128	147.2303
4.266667	128	139.5378	155.6634	127.0958	141.3796

Swim Example – 20221010-0002

Time	Index	Head Orientation	Whisker 1	Whisker 2	mean
0.033333	1	123.5697			
0.066667	2	124.7213			
0.1	3	119.7976			
0.133333	4	121.4487			
0.166667	5	118.3274			
0.2	6	117.5042			
0.233333	7	120.8946			
0.266667	8	119.3353			
0.3	9	113.3477	52.03382	50.37853	51.20617
0.333333	10	116.9395	57.26313	53.50458	55.38386
0.366667	11	115.0915	50.76835	50.10842	50.43838
0.4	12	113.0913	44.64837	53.43454	49.04146
0.433333	13	113.0436	45.71494	52.0982	48.90657
0.466667	14	111.2716	45.40001	46.04675	45.72338
0.5	15	109.8321	42.95298	48.96642	45.9597
0.533333	16	112.6865	44.48794	51.54219	48.01506
0.566667	17	109.4626	35.89187	44.54616	40.21901
0.6	18	113.7948	44.43238	46.6685	45.55044
0.633333	19	113.2526	39.60841	46.68128	43.14485
0.666667	20	113.0913	38.94322	42.99504	40.96913
0.7	21	112.5206	36.39025	44.63001	40.51013
0.733333	22	115.8444	38.30932	47.34582	42.82757
0.766667	23	119.0546	44.02748	50.71059	47.36904
0.8	24	112.4259	31.70941	41.37237	36.54089
0.833333	25	117.6632	36.27889	48.76808	42.52348
0.866667	26	115.641	33.77111	43.04371	38.40741
0.9	27	118.8627	38.02507	44.34136	41.18322
0.933333	28	118.2174	36.34746	44.65774	40.5026
0.966667	29	121.1757	35.33534	39.47285	37.40409
1	30	126.0572	43.52881	44.35439	43.9416
1.033333	31	125.6764	44.16535	49.71265	46.939
1.066667	32	125.967	41.97604	43.97176	42.9739

Swoop Example – 20220622-0001

Time	Index	Head Orientation	Head Or	Whisker 1
0.033333	1	161.3950345	161.3950345	
0.066667	2	163.4126614	163.4126614	
0.1	3	166.4834319	166.4834319	
0.133333	4	169.8949636	169.8949636	
0.166667	5	171.9455856	171.9455856	
0.2	6	172.4761796	172.4761796	
0.233333	7	178.1671605	178.1671605	
0.266667	8	178.8982939	178.8982939	
0.3	9	174.075418	174.075418	
0.333333	10	163.8865818	163.8865818	
0.366667	11	162.7185016	162.7185016	
0.4	12	161.17529	161.17529	
0.433333	13	165.4257838	165.4257838	
0.466667	14	170.1120112	170.1120112	
0.5	15	173.2599439	173.2599439	
0.533333	16	173.2599439	173.2599439	
0.566667	17	173.2599439	173.2599439	
0.6	18	177.9174347	177.9174347	
0.633333	19	-171.740562	171.740562	
0.666667	20	-178.5185428	178.5185428	
0.7	21	-179.0371364	179.0371364	
0.733333	22	-170.9492783	170.9492783	
0.766667	23	-170.4554744	170.4554744	
0.8	24	-160.5069381	160.5069381	95.45682
0.833333	25	-165.379126	165.379126	86.18593
0.866667	26	-158.025492	158.025492	74.141
0.9	27	-159.1022345	159.1022345	77.49516
0.933333	28	-149.9103138	149.9103138	67.63214
0.966667	29	-149.4207731	149.4207731	66.67397
1	30	-149.1115335	149.1115335	74.68408
1.033333	31	-146.1951114	146.1951114	66.75612
1.066667	32	-140.3470816	140.3470816	61.65701
1.1	33	-148.0850599	148.0850599	67.395
1.133333	34	-146.1853766	146.1853766	65.50897
1.166667	35	-149.3002774	149.3002774	72.35552
1.2	36	-142.9434718	142.9434718	64.66858
1.233333	37	-137.7554247	137.7554247	60.28423
1.266667	38	-133.983022	133.983022	54.86855
1.3	39	-134.0398254	134.0398254	59.38996
1.333333	40	-135.9710219	135.9710219	66.52707
1.366667	41	-138.6138808	138.6138808	72.75942

1.4	42	-136.8087393	136.8087393	66.34539
1.433333	43	-135.9821172	135.9821172	64.04259
1.466667	44	-134.683452	134.683452	61.61097
1.5	45	-135.6437457	135.6437457	59.09236
1.533333	46	-135.3350592	135.3350592	59.3713
1.566667	47	-135.9601746	135.9601746	60.26045
1.6	48	-134.683452	134.683452	61.3827
1.633333	49	-128.3087356	128.3087356	58.36483
1.666667	50	-121.6672164	121.6672164	51.83787
1.7	51	-128.6598083	128.6598083	59.35526
1.733333	52	-125.9229462	125.9229462	59.27851
1.766667	53	-123.4165814	123.4165814	58.56137
1.8	54	-122.5857074	122.5857074	60.88646
1.833333	55	-120.3932626	120.3932626	59.00372
1.866667	56	-117.5360731	117.5360731	
1.9	57	-118.2374455	118.2374455	
1.933333	58	-116.3618755	116.3618755	
1.966667	59	-117.6459754	117.6459754	
2	60	-117.5880814	117.5880814	
2.033333	61	-117.3635469	117.3635469	
2.066667	62	-116.9878906	116.9878906	
2.1	63	-115.9188856	115.9188856	
2.133333	64	-117.1811111	117.1811111	
2.166667	65	-121.9001569	121.9001569	
2.2	66	-115.9533757	115.9533757	
2.233333	67	-119.7448813	119.7448813	
2.266667	68	-119.8989018	119.8989018	
2.3	69	-121.5513849	121.5513849	
2.333333	70	-123.8048886	123.8048886	
2.366667	71	-124.01935	124.01935	
2.4	72	-126.1581854	126.1581854	
2.433333	73	-131.4032829	131.4032829	
2.466667	74	-135.6662	135.6662	
2.5	75	-141.7889746	141.7889746	
2.533333	76	-145.2108677	145.2108677	

Supplementary Data 3: Featured audio trace

time	Signal amp
0	0.015308
0.005	0.028793
0.01	0.032078
0.015	0.006668
0.02	0.006981
0.025	0.099019
0.03	-0.03613
0.035	-0.01363
0.04	0.126267
0.045	0.074145
0.05	-0.01308
0.055	0.147423
0.06	0.170109
0.065	0.068664
0.07	0.156105
0.075	0.128757
0.08	0.144994
0.085	0.207779
0.09	0.042417
0.095	0.07809
0.1	0.091392
0.105	-0.08891
0.11	-0.06651
0.115	-0.07691
0.12	-0.26967
0.125	-0.27489
0.13	-0.22952
0.135	-0.2087
0.14	-0.22494
0.145	-0.14337
0.15	-0.1023
0.155	-0.05331
0.16	0.028008
0.165	0.053258
0.17	0.137593
0.175	0.157078
0.18	0.154534
0.185	0.104679
0.19	0.04886
0.195	0.074462
0.2	-0.02164

0.205	-0.08098
0.21	-0.02925
0.215	-0.06196
0.22	-0.09807
0.225	-0.03172
0.23	0.067624
0.235	0.014622
0.24	0.01577
0.245	0.010883
0.25	0.000204
0.255	0.091615
0.26	0.069868
0.265	-0.06351
0.27	0.036399
0.275	-0.05083
0.28	-0.08659
0.285	-0.01314
0.29	-0.04133
0.295	-0.03993
0.3	-0.00418
0.305	0.029675
0.31	-0.01493
0.315	0.004048
0.32	0.029824
0.325	0.025435
0.33	0.051769
0.335	-0.04349
0.34	-0.00745
0.345	0.001167
0.35	0.006529
0.355	-0.04478
0.36	-0.00493
0.365	0.048885
0.37	-0.00816
0.375	-0.02235
0.38	0.005703
0.385	0.025274
0.39	0.03699
0.395	0.006569
0.4	-0.0058
0.405	0.026747
0.41	-0.00957
0.415	-0.01716
0.42	0.014489

0.425	-0.01616
0.43	0.00536
0.435	-0.0038
0.44	0.006467
0.445	0.031454
0.45	0.016962
0.455	0.018191
0.46	0.020642
0.465	0.001785
0.47	0.017023
0.475	0.057788
0.48	0.068664
0.485	0.038231
0.49	0.011976
0.495	-0.00794
0.5	0.046775
0.505	0.01505
0.51	0.005859
0.515	0.016008
0.52	-0.09549
0.525	-0.08342
0.53	-0.02414
0.535	-0.06794
0.54	-0.11623
0.545	-0.04732
0.55	-0.04713
0.555	-0.10952
0.56	0.022218
0.565	0.040946
0.57	-0.03604
0.575	-0.01976
0.58	0.064456
0.585	0.013831
0.59	0.009372
0.595	0.034083
0.6	0.026198
0.605	-0.01051
0.61	0.001933
0.615	-0.02815
0.62	0.003184
0.625	-0.02997
0.63	-0.01032
0.635	0.036093
0.64	-0.0496

0.645	0.019756
0.65	-0.01604
0.655	0.017734
0.66	-0.00369
0.665	0.015703
0.67	-0.01836
0.675	-0.05159
0.68	0.057176
0.685	-0.02717
0.69	-0.01341
0.695	0.008166
0.7	0.013
0.705	-0.00065
0.71	0.012473
0.715	0.05004
0.72	0.017813
0.725	-0.05272
0.73	-0.00358
0.735	0.007155
0.74	-0.02986
0.745	0.003952
0.75	0.040353
0.755	-0.02047
0.76	-0.0077
0.765	-0.00344
0.77	0.052876
0.775	0.053178
0.78	-0.03021
0.785	-0.05286
0.79	0.032717
0.795	0.022275
0.8	-0.04148
0.805	0.042782
0.81	0.014777
0.815	-0.00679
0.82	-0.03292
0.825	0.009458
0.83	0.003249
0.835	-0.05053
0.84	-0.02133
0.845	-0.09072
0.85	-0.01566
0.855	0.026704
0.86	0.034676

0.865	0.04651
0.87	0.108972
0.875	0.117987
0.88	0.06571
0.885	0.06552
0.89	0.073288
0.895	0.061888
0.9	-0.01074
0.905	-0.09688
0.91	-0.05352
0.915	-0.11804
0.92	-0.13772
0.925	-0.15014
0.93	-0.12804
0.935	-0.10829
0.94	-0.07528
0.945	0.014129
0.95	0.025663
0.955	0.117949
0.96	0.086382
0.965	0.10379
0.97	0.077476
0.975	0.115661
0.98	0.09003
0.985	0.004752
0.99	-0.03082
0.995	-0.0709
1	-0.00516
1.005	-0.04298
1.01	-0.07826
1.015	-0.01511
1.02	-0.01893
1.025	0.00838
1.03	-0.00406
1.035	0.038105
1.04	0.087264
1.045	0.034381
1.05	-0.00616
1.055	-0.00333
1.06	0.048356
1.065	-0.03399
1.07	-0.04748
1.075	0.020022
1.08	-0.06472

1.085	-0.06395
1.09	0.040264
1.095	-0.00298
1.1	0.026913
1.105	-0.00966
1.11	-0.01482
1.115	0.015805
1.12	0.024198
1.125	0.021487
1.13	-0.05148
1.135	-0.03001
1.14	-0.0017
1.145	0.028965
1.15	-0.00055
1.155	-0.02342
1.16	0.008706
1.165	0.012347
1.17	0.041183
1.175	0.033952
1.18	-0.00907
1.185	0.019212
1.19	0.023192
1.195	0.008591
1.2	0.046405
1.205	-0.01613
1.21	-0.00817
1.215	0.010559
1.22	0.039239
1.225	0.003421
1.23	-0.00962
1.235	-0.00683
1.24	-0.01276
1.245	0.007878
1.25	0.049728
1.255	-8.09E-05
1.26	-0.00238
1.265	0.038621
1.27	0.022391
1.275	0.007906
1.28	0.012616
1.285	-0.01132
1.29	-0.01249
1.295	-0.02205
1.3	-0.04126

1.305	-0.05184
1.31	-0.0939
1.315	-0.13513
1.32	-0.0613
1.325	-0.00918
1.33	0.019731
1.335	0.065604
1.34	0.039026
1.345	0.094281
1.35	0.215217
1.355	0.097787
1.36	0.057423
1.365	0.072116
1.37	0.006001
1.375	-0.026
1.38	-0.05117
1.385	-0.07905
1.39	-0.14651
1.395	-0.14971
1.4	-0.1432
1.405	-0.17462
1.41	-0.09489
1.415	-0.06194
1.42	0.023531
1.425	0.083861
1.43	0.088376
1.435	0.168756
1.44	0.144572
1.445	0.127169
1.45	0.075711
1.455	0.06549
1.46	0.051809
1.465	-0.03231
1.47	-0.10156
1.475	-0.05475
1.48	-0.01279
1.485	-0.00588
1.49	-0.03158
1.495	-0.03486
1.5	0.039014
1.505	0.033638
1.51	0.015295
1.515	0.071378
1.52	0.017588

1.525	0.012085
1.53	-0.03666
1.535	-0.04088
1.54	-0.04565
1.545	-0.02115
1.55	-0.01481
1.555	-0.0616
1.56	-0.00534
1.565	-0.01227
1.57	-0.00296
1.575	0.076734
1.58	0.025624
1.585	0.012352
1.59	0.002489
1.595	-0.02011
1.6	0.016311
1.605	0.012959
1.61	-0.01642
1.615	-0.02967
1.62	-0.00794
1.625	-0.01072
1.63	-0.04116
1.635	0.036529
1.64	0.047257
1.645	0.01082
1.65	0.019778
1.655	-0.00703
1.66	0.053691
1.665	-0.02428
1.67	-0.01739
1.675	0.049136
1.68	-0.05994
1.685	-0.02023
1.69	-0.02855
1.695	0.030858
1.7	-0.01986
1.705	-0.13226
1.71	0.016143
1.715	-0.02802
1.72	-0.07487
1.725	-0.02407
1.73	0.016217
1.735	-0.00172
1.74	-0.04729

1.745	0.040308
1.75	-0.05893
1.755	-0.02326
1.76	0.021491
1.765	-0.0279
1.77	-0.02777
1.775	-0.09543
1.78	-0.08409
1.785	-0.0788
1.79	-0.02985
1.795	0.020892
1.8	0.016443
1.805	0.050619
1.81	0.088636
1.815	0.097359
1.82	0.145851
1.825	0.112289
1.83	0.035321
1.835	-0.00928
1.84	-0.04215
1.845	-0.14925
1.85	-0.1791
1.855	-0.16695
1.86	-0.16176
1.865	-0.22231
1.87	-0.12893
1.875	-0.05491
1.88	-0.00757
1.885	0.06461
1.89	0.088348
1.895	0.160966
1.9	0.191711
1.905	0.137511
1.91	0.138011
1.915	0.043194
1.92	-0.06758
1.925	-0.00387
1.93	-0.00978
1.935	-0.09899
1.94	-0.05473
1.945	-0.03149
1.95	-0.07018
1.955	0.027748
1.96	0.08423

1.965	0.029239
1.97	0.059499
1.975	0.072509
1.98	0.031423
1.985	0.028
1.99	-0.02018
1.995	-0.0629
2	0.041689
2.005	-0.00373
2.01	-0.06391
2.015	-0.00571
2.02	-0.05421
2.025	-0.05708
2.03	0.054466
2.035	0.040672
2.04	0.018541
2.045	0.048763
2.05	-0.03736
2.055	0.045891
2.06	0.020369
2.065	-0.00672
2.07	0.002747
2.075	-0.04355
2.08	-0.00139
2.085	0.037132
2.09	0.000329
2.095	0.03821
2.1	0.037134
2.105	-0.03454
2.11	0.04416
2.115	0.008772
2.12	-0.00275
2.125	-0.00652
2.13	0.053965
2.135	0.045739
2.14	-0.02795
2.145	-0.02988
2.15	-0.00505
2.155	0.015393
2.16	-0.02097
2.165	-0.01311
2.17	-0.0299
2.175	0.022921
2.18	0.051504

2.185	-0.01823
2.19	0.007945
2.195	0.006196
2.2	0.014196
2.205	-0.00187
2.21	-0.00442
2.215	-0.00059
2.22	-0.01572
2.225	-0.07822
2.23	-0.12856
2.235	-0.06469
2.24	-0.07646
2.245	-0.05936
2.25	-0.08232
2.255	0.037677
2.26	0.161893
2.265	0.11555
2.27	0.156936
2.275	0.175774
2.28	0.155035
2.285	0.07457
2.29	0.080919
2.295	0.047958
2.3	-0.12811
2.305	-0.12057
2.31	-0.10494
2.315	-0.29145
2.32	-0.27526
2.325	-0.12804
2.33	-0.16107
2.335	-0.08569
2.34	0.054552
2.345	0.118657
2.35	0.215239
2.355	0.208875
2.36	0.212083
2.365	0.235312
2.37	0.117221
2.375	0.000709
2.38	-0.00163
2.385	-0.08851
2.39	-0.1658
2.395	-0.08053
2.4	-0.08754

2.405	-0.06728
2.41	0.029692
2.415	-0.00322
2.42	0.077311
2.425	0.024435
2.43	0.034268
2.435	0.0415
2.44	-0.00437
2.445	0.016455
2.45	-0.01534
2.455	-0.07615
2.46	-0.11198
2.465	-0.03698
2.47	-0.07299
2.475	-0.05936
2.48	0.001688
2.485	-0.05156
2.49	0.043153
2.495	0.099384
2.5	-0.04239
2.505	0.006335
2.51	0.071557
2.515	0.009794
2.52	0.021028
2.525	-0.03727
2.53	-0.06252
2.535	0.009862
2.54	-0.04907
2.545	-0.00098
2.55	0.01033
2.555	-0.04437
2.56	0.004138
2.565	0.017346
2.57	0.007442
2.575	0.086161
2.58	0.039605
2.585	-0.03791
2.59	-0.03209
2.595	0.031473
2.6	-0.01974
2.605	-0.07376
2.61	0.007263
2.615	0.012122
2.62	-0.04512

2.625	-0.01999
2.63	-0.01043
2.635	-0.01577
2.64	0.034729
2.645	-0.01221
2.65	0.009511
2.655	0.025291
2.66	-0.03433
2.665	0.01344
2.67	0.05032
2.675	-0.02051
2.68	-0.07426
2.685	-0.00912
2.69	-0.06869
2.695	-0.0636
2.7	0.050203
2.705	0.055686
2.71	0.130215
2.715	0.227661
2.72	0.221564
2.725	0.162848
2.73	0.17168
2.735	0.115462
2.74	0.029264
2.745	-0.01661
2.75	-0.14406
2.755	-0.21637
2.76	-0.30814
2.765	-0.33866
2.77	-0.27807
2.775	-0.29657
2.78	-0.20368
2.785	-0.01427
2.79	0.036808
2.795	0.140559
2.8	0.371994
2.805	0.328171
2.81	0.273822
2.815	0.335721
2.82	0.149116
2.825	-0.00027
2.83	-0.04098
2.835	-0.10069
2.84	-0.1259

2.845	-0.09473
2.85	-0.14753
2.855	-0.02363
2.86	0.038098
2.865	0.051808
2.87	0.109872
2.875	0.078348
2.88	0.103409
2.885	0.085274
2.89	0.020538
2.895	-0.04074
2.9	-0.06316
2.905	-0.11401
2.91	-0.10377
2.915	-0.08248
2.92	-0.08975
2.925	-0.11387
2.93	-0.07795
2.935	0.052313
2.94	0.03118
2.945	0.063545
2.95	0.067409
2.955	-0.02414
2.96	0.059237
2.965	0.053625
2.97	-0.02724
2.975	-0.04246
2.98	-0.02364
2.985	0.027633
2.99	-0.03578
2.995	-0.06795
3	0.007452
3.005	0.004952
3.01	0.006418
3.015	0.070106
3.02	0.097072
3.025	0.017581
3.03	-0.04527
3.035	0.005565
3.04	-0.01854
3.045	-0.03643
3.05	-0.02387
3.055	-0.02922
3.06	-0.07414

3.065	-0.0671
3.07	0.008278
3.075	0.002158
3.08	0.005925
3.085	0.041367
3.09	-0.00412
3.095	0.091737
3.1	0.013423
3.105	0.046302
3.11	0.070359
3.115	-0.01017
3.12	0.001242
3.125	-0.00121
3.13	0.015404
3.135	-0.02633
3.14	-0.05663
3.145	-0.02221
3.15	0.049628
3.155	0.136943
3.16	0.170932
3.165	0.220087
3.17	0.230696
3.175	0.308026
3.18	0.249095
3.185	0.047124
3.19	0.048012
3.195	-0.08217
3.2	-0.23775
3.205	-0.26671
3.21	-0.36316
3.215	-0.42331
3.22	-0.35029
3.225	-0.32191
3.23	-0.24732
3.235	-0.03398
3.24	0.081816
3.245	0.239409
3.25	0.42611
3.255	0.332249
3.26	0.320282
3.265	0.345056
3.27	0.158652
3.275	0.032487
3.28	-0.05845

3.285	-0.14839
3.29	-0.14918
3.295	-0.20128
3.3	-0.11742
3.305	-0.00909
3.31	0.011598
3.315	0.045479
3.32	0.120003
3.325	0.127907
3.33	0.083898
3.335	0.07554
3.34	0.026411
3.345	-0.03955
3.35	-0.08048
3.355	-0.13265
3.36	-0.12157
3.365	-0.16271
3.37	-0.16019
3.375	-0.08178
3.38	-0.05693
3.385	-0.00455
3.39	0.05017
3.395	0.060332
3.4	0.039918
3.405	0.040043
3.41	0.059282
3.415	0.002335
3.42	-0.00642
3.425	-0.03124
3.43	-0.07247
3.435	-0.05409
3.44	-0.06128
3.445	-0.06766
3.45	-0.02271
3.455	0.022245
3.46	0.026207
3.465	0.009909
3.47	0.044127
3.475	-0.01863
3.48	0.018523
3.485	0.082281
3.49	-0.06548
3.495	-0.05136
3.5	-0.08315

3.505	-0.08441
3.51	0.016223
3.515	-0.02837
3.52	-0.05245
3.525	0.032438
3.53	-0.01559
3.535	0.021915
3.54	0.051617
3.545	0.02323
3.55	-0.04406
3.555	-0.0146
3.56	-0.03981
3.565	-0.04011
3.57	0.052005
3.575	0.091692
3.58	0.208297
3.585	0.227084
3.59	0.269258
3.595	0.274157
3.6	0.135315
3.605	0.115028
3.61	-0.0368
3.615	-0.0672
3.62	-0.23805
3.625	-0.35746
3.63	-0.3914
3.635	-0.41368
3.64	-0.37921
3.645	-0.32129
3.65	-0.12143
3.655	0.101536
3.66	0.234967
3.665	0.361762
3.67	0.482524
3.675	0.420208
3.68	0.352986
3.685	0.206509
3.69	0.001565
3.695	-0.03657
3.7	-0.16252
3.705	-0.23162
3.71	-0.23611
3.715	-0.19315
3.72	-0.12546

3.725	-0.03418
3.73	0.007597
3.735	0.014514
3.74	0.113696
3.745	0.094422
3.75	0.033058
3.755	0.077278
3.76	0.030635
3.765	-0.07457
3.77	-0.10731
3.775	-0.13526
3.78	-0.16523
3.785	-0.21333
3.79	-0.10323
3.795	-0.09956
3.8	-0.05887
3.805	0.033053
3.81	0.057303
3.815	0.06257
3.82	0.067365
3.825	0.040647
3.83	0.061481
3.835	0.053151
3.84	-0.03222
3.845	-0.09392
3.85	-0.05339
3.855	-0.01901
3.86	-0.10986
3.865	-0.05923
3.87	-0.07021
3.875	-0.06148
3.88	0.039825
3.885	-0.00219
3.89	0.056898
3.895	-0.00681
3.9	-0.0179
3.905	-0.02151
3.91	-0.02929
3.915	-0.01297
3.92	-0.08619
3.925	-0.07743
3.93	-0.05716
3.935	-0.06452
3.94	-0.03967

3.945	0.040751
3.95	0.062451
3.955	0.051994
3.96	0.049914
3.965	0.006905
3.97	0.024909
3.975	0.090508
3.98	0.047314
3.985	-0.0537
3.99	-0.00146
3.995	-0.05427
4	-0.0858
4.005	-0.06037
4.01	-0.12857
4.015	-0.06255
4.02	-0.05625
4.025	-0.00326
4.03	0.028684
4.035	0.036311
4.04	0.090372
4.045	0.100505
4.05	0.09123
4.055	0.032851
4.06	0.043448
4.065	0.010109
4.07	-0.00161
4.075	-0.01484
4.08	-0.03545
4.085	-0.0041
4.09	-0.0339
4.095	0.000773
4.1	0.01255
4.105	0.001079
4.11	0.01456
4.115	0.0034
4.12	0.040899
4.125	0.017027
4.13	-0.02566
4.135	-0.03164
4.14	-0.02855
4.145	-0.04269
4.15	-0.08738
4.155	-0.00641
4.16	-0.04335

4.165	-0.08703
4.17	-0.03029
4.175	-0.02389
4.18	-0.00846
4.185	-0.0006
4.19	-0.04106
4.195	0.02479
4.2	0.077644
4.205	0.037931
4.21	-0.06381
4.215	-0.03506
4.22	0.042984
4.225	0.035197
4.23	-0.005
4.235	-0.02253
4.24	0.019034
4.245	-0.04628
4.25	0.048806
4.255	0.066474
4.26	-0.00474
4.265	-0.02289
4.27	0.009797
4.275	-0.03623
4.28	0.040152
4.285	0.048336
4.29	-0.0867
4.295	0.01301
4.3	-0.02043
4.305	-0.08251
4.31	0.069054
4.315	-0.04656
4.32	-0.05562
4.325	0.008979
4.33	-0.04832
4.335	0.038575
4.34	0.028015
4.345	-0.00573
4.35	0.017091
4.355	-0.01006
4.36	-0.05798
4.365	0.040527
4.37	-0.04431
4.375	-0.02759
4.38	0.053228

4.385	0.017318
4.39	0.048999
4.395	-0.01364
4.4	-0.0291
4.405	0.00056
